# How can we support the individual breastfeeding experience? Quantitative results from a mixed-methods study

**DOI:** 10.1186/s13006-025-00726-4

**Published:** 2025-05-17

**Authors:** Theresa Philomena Ertlmaier, Oana Costea, Anja Borgmann-Staudt, Monika Berns, Georg Weikert, Mathilde Kersting, Magdalena Balcerek

**Affiliations:** 1 Department of Pediatric Oncology and Hematology, Charité-Universitätsmedizin Berlin, corporate member of, Freie Universität Berlin and Humboldt Universität Zu Berlin, Augustenburger Platz 1, Berlin, 13353 Germany; 2 Department of Gynaecology and Obstetrics, Gemeinschaftskrankenhaus Havelhöhe, Kladower Damm 221, Berlin, 14089 Germany; 3 Department of Neonatology, Charité-Universitätsmedizin Berlin, corporate member of, Freie Universität Berlin and Humboldt Universität Zu Berlin, Augustenburger Platz 1, Berlin, 13353 Germany; 4 Department of Neonatology, DRK Kliniken Berlin Westend, Spandauer Damm 130, Berlin, 14050 Germany; 5 Research Department of Child Nutrition, University Hospital of Pediatrics and Adolescent Medicine, St. Josef-Hospital, Ruhr-University, Bochum, Germany

**Keywords:** Breastfeeding, Duration, Influencing factors, Maternal comfort, Baby-friendly hospital, Anthroposophy

## Abstract

**Background:**

Despite the health benefits of breastfeeding only 57% of infants were exclusively breastfed at 4 months postpartum in Germany in 2017–2019. Due to the gap between the actual exclusive breastfeeding (EBF) rates and recommendations, we aimed at investigating further factors influencing breastfeeding duration.

**Methods:**

This prospective observational study conducted in Berlin, Germany from 11/2022–05/2024 implemented a mixed-methods design with concurrent triangulation. We present quantitative results here. First-time mothers were surveyed at birth and 2, 6 and 12 months postpartum. Breastfeeding status was assessed by asking “How are you currently feeding your child?” and, if anything other than EBF (defined as supply of breastmilk without liquids or solids) was indicated, “I breastfed exclusively until [date]”. Maternal perception was assessed by asking “How comfortable do you currently feel with breastfeeding/feeding your child?”, with comfort referring to well-being/statisfaction/feeling good (German = *wohlfühlen*). Obstetric and newborn characteristics were collected from recruiting hospital’s health records: an anthroposophic baby-friendly certified hospital (a-BF), a baby-friendly certified hospital (BF) and a university hospital not certified as baby-friendly (non-BF). Data were analysed descriptively and through multivariate analysis.

**Results:**

Most of the 326 participating mothers had initiated breastfeeding in the delivery room (94.7%). Mothers reported EBF for a median duration of 5.7 months, with 76.6% achieving ≥ 4 months. High levels of maternal comfort with breastfeeding 2 months postpartum were significantly associated with an EBF duration ≥ 4 months (aOR 7.25, CI 95% 2.11, 24.9). An intended EBF duration of 4–7 months (aOR 4.08, CI 95% 0.29, 57.77), higher breastfeeding comfort shortly after birth (aOR 1.79, CI 95% 0.49, 6.59), delivery in the a-BF clinic (aOR 1.59, CI 95% 0.41, 6.14) and high satisfaction regarding breastfeeding support in the hospital (aOR 1.39, CI 95% 0.41, 4.70) increased likeliness of EBF ≥ 4 months.

**Conclusion:**

Our results emphasize the pivotal role of the mother’s comfort in the breastfeeding process and it’s impact on breastfeeding duration. Strategies to enhace maternal comfort therefore need to be specifically included in maternal care. To explore key aspects of maternal comfort qualitative interviews will address the individual experiences in the breastfeeding journey and identify parent-centred strategies for sustainable breastfeeding.

**Supplementary Information:**

The online version contains supplementary material available at 10.1186/s13006-025-00726-4.

## Background

The immediate and long-term importance of breastfeeding for the health of both mother and child have been well documented [1–4]; the breastfeeding duration being decisive for the preventive effect. Accordingly, the World Health Organization (WHO) and the United Nations International Children's Emergency Fund (UNICEF) recommend exclusive breastfeeding for 6 months and continued breastfeeding along with introducing complementary foods until 2 years of age or longer [5]. In Germany, the National Breastfeeding Commission recommends exclusive breastfeeding for 4 to 6 months and beyond accompanying the introduction of solid foods [6].

Factors associated with longer breastfeeding durations include: higher maternal age, normal pre-pregnancy body-mass index (BMI), non-smoking during pregnancy, term delivery, vaginal birth, normal weight and female infants; as well as socio-cultural factors such as maternal intention to breastfeed, higher educational level, no pacifier utilization, co-sleeping and feeling comfortable breastfeeding in public. Breastfeeding duration can be enhanced by medical interventions including antenatal classes, early postpartum breastfeeding and avoidance of supplementary feeding in hospital [7–10].

Several of these factors are considered part of the 10 steps–the baby-friendly way–formulated by the baby-friendly hospital initiative of the WHO and UNICEF in 1991 to sustainably support breastfeeding rates and duration [11]. Implementing these 10 steps positively effects the short, medium and long-term breastfeeding outcomes [12]. Hospitals can be certified as baby-friendly, but there are differences in the level of compliance with the guidelines [13]. Certain hospitals also specialise in anthroposophic medicine wich “is an integrative system that improves health outcomes through a holistic approach to treatment that includes physical, psychological and social health” [14]. In obstetrics, this can mean, amongst others, that families are supported on their individual journey in a cosy atmosphere [15].

Although breastfeeding rates in Germany have come closer to the recommendations over the last 20 years, only 40% of infants were exclusively breastfed at 4 months postpartum in 2012–2016 [16] and 57% in 2017–2019 [17]. In these studies, mothers completed questionnaires about their child's current nutrition with exclusively breastfeeding defined as the supply of breastmilk or human milk without any additional liquids or solids except medicine, vitamin, mineral drops or syrups.

Due to the discrepancy between the actual breastfeeding rates and the recommendations, this study was conducted to identify further influencing factors that increase the number of mothers breastfeeding for longer.

## Methods

### Aim

Our mixed-method study aimed to explore influencing factors on the breastfeeding duration, specifically focussing on the individual breastfeeding experience to identify approaches from the parental perspective for a sustainable breastfeeding process. We here present results from the quantitative part of the study.

### Study design and setting

From November 2022 to May 2024, this prospective observational study explored the longitudinal breastfeeding experience of women who gave birth in three different hospital settings in Berlin, Germany: a university hospital not certified as baby-friendly (non-BF), a baby-friendly (BF) certified hospital and an anthroposophic baby-friendly (a-BF) certified hospital. These hospitals also differ regarding their birth numbers, caesarean section rates and perinatal centre levels: In the non-BF hospital (certified as a level 1 perinatal centre [18]) 3205 births were documented in 2023, of which 25–30% were caesarean sections; as well as 2414 births with 37% caesarean sections in the BF hospital (level 2 [19]) and 1141 births with 21% caesarean sections in the a-BF hospital which does not include a specific neonatal care unit. This mixed-methods study is designed convergently according to the concurrent triangulation [20]. A staged approach to interpretation and reporting was chosen to achieve the connecting approach of our methodology. Supplemental interviews were conducted with a subset of mothers from this larger sample and is subject of future publications. We here present the results of the quantitative part.

Ethical approval was granted by the ethical board (EA2/105/22) and the study was registered in the central study register (3,000,616) of the Charité-Universitätsmedizin Berlin.

### Sample

Eligible participants were primipara mothers who delivered term newborns (gestational age ≥ 37+0 weeks) in one of the participating hospitals during the survey period and who were cared for with their newborns in the postnatal ward at recruitment. Only primiparous mothers were included to avoid a possible influence of previous breastfeeding experience. A significant postnatal mother–child separation requiring transfer to the intensive care unit was considered an exclusion criterion. Multiple births were not excluded to achieve a more representative population. Additionally, mothers had to be ≥18 years, have sufficient German proficiency, an internet access and provide written informed consent. A sample size was calculated based on previous studies on the primary outcome–breastfeeding rates at 4 months postpartum–for orientation purposes for this exploratory study. A reasonable confidence interval of [0.49, 0.61] could be achieved with a case number of 250 mothers, whereas inclusion of 450 mothers would result in a confidence interval of [0.51, 0.60]. In total, we achieved to approach 377 mothers during the study period.

### Data collection

**Fig. 1:**
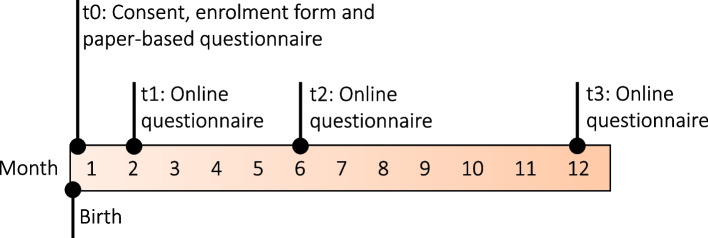
Study design: recruitment and survey time points (t)

Recruitment took place in the postnatal wards of participating hospitals from November 2022 to April 2023 and was conducted in collaboration with the study’s cooperation partners accommodating their availabilities and working schedules. Eligible mothers were informed about the study verbally and in writing the first days after birth and were asked to complete an initial, brief paper-based questionnaire on-site (t0). Maternal and newborn characteristics, including obstetrical and socio-demographic data, were collected from hospital health records at t0. Follow-up online questionnaires (SoSci Survey) exploring the breastfeeding experience were distributed via e-mail link after 2 (t1), 6 (t2) and 12 (t3) months postpartum (Fig. 1). Reminders were sent after 1 and 3 weeks. All study data was collected pseudonymised.

Questionnaire information was considered if it had been completed before the follow-up questionnaire was distributed. Only mothers, who reported ongoing breastfeeding received the follow-up questionnaire.

### Measurement

The questionnaires were developed specifically for this study and tested by staff on the postnatal wards before being applied due to a lack of research findings with questionnaires relating to individual breastfeeding experiences. The current breastfeeding status was recorded according to the definitions of the German National Breastfeeding Commission (exclusive, partial, no longer or no breastfeeding [21]). Exclusive breastfeeding was defined as currently not giving liquids, infant formula or complementary foods in addition to breastmilk. Breast feeding status was assessed by the following question:

How are you currently feeding your child?

- I exclusively breastfeed with breastmilk.
- I breastfeed and supplement with fluids/formula/complementary food.
- I stopped breastfeeding since [date].
- I have never breastfed.

If mothers reported other than exclusive breastfeeding, they were asked to answer the following: “I breastfed exclusively until [date]”. The breastfeeding experience was assessed through questions on the intended duration of exclusive breastfeeding (open response), reasons for breastfeeding or no more/not breastfeeding (Likert scale), use of breastfeeding aids (multiple choice), as well as the mother’s motivation (Likert scale), comfort (German: “*wohlfühlen*”; relating to feeling good about/well-being/satisfied/comfort with breastfeeding process and situation, Likert scale), and perception of breastfeeding (Likert scale). Partner’s attitudes (single choice) and involvement (multiple choice) were also documented. Breastfeeding counselling in the hospital was examined, including method (multiple choice), satisfaction (Likert scale), timing (multiple choice) and additional information sources (multiple choice), as well as reasons for the choice of maternity clinic (multiple choice). The 5-point Likert scale was used. In the follow-up at t2 and t3, the same questions were asked as in t1 regarding the current breastfeeding status, perception of breastfeeding, reasons for breastfeeding, no more or not breastfeeding and, if applicable, questions on the process of ending breastfeeding and breastfeeding aids (e.g. lactation tea, supplemental nursing system, wool wax, breast caps), the involvement and role of the partner, and whether the mother is currently working. Socio-economic data were collected via online questionnaire at t1 to reduce the survey burden shortly after having given birth (t0). This information covered the perceived socio-economic status assessed by the German MacArthur scale [22], migration background (self or parent born outside Germany [23]), newborn’s living situation, and employment status of both parents. An overview over questions included in the different questionnaires are presented in Additional file 1.

Maternal information collected from health records included age at time of birth, alcohol consumption and smoking during pregnancy as well as pre-pregnancy BMI. Obstetric details included information on the maternity clinic, multiple births, birth mode, use of peridural/epidural anaesthesia, initiation of breastfeeding in the delivery room, postpartum separation, rooming-in and discharge dates. For assessment of the newborn’s characteristics sex, weight, length, Apgar scores at 5 and 10 minutes and postnatal pH-value were documented.

### Data analysis

Statistical analyses were conducted using IBM® SPSS® Statistics Version 29 and Microsoft® Excel Version 16 for Mac. Data were analysed descriptively with absolute (number, median and range, mean and standard deviation) and relative frequencies, calculated on the bases of cases with complete data. Breastfeeding duration was categorized according to the recommendations of the German National Breastfeeding Commission for an exclusive breastfeeding duration (≥ 4 months; < 4 months) [6]. Open responses regarding the intended duration of exclusive breastfeeding were categorised and analysed descriptively. Further open responses are analysed qualitatively and will be part of further publications. Statistical significance was analysed according to standard practice using the t-test for independent samples, chi-square-test, Mann–Whitney U-test or two-factor variance analysis for ranks according to Friedman. The significance level is 5%. Multivariate analysis was carried out using binary logistic regression considering a confidence interval of 95%. A combination of established factors identified from current literature, including maternal age and birth weight, newborn sex, birth mode [7, 10, 24], as well as innovative factors arising from the descriptive analysis, including type of maternity clinic, intended breastfeeding duration, active breastfeeding counselling, as well as perception of breastfeeding-initiation support at t0 and comfort concerning breastfeeding/feeding at t0 and t1, was selected. The data is presented in compliance with the STROBE guidelines.

## Results

### Participant characteristics and questionnaire completion

Overall, 326 of 377 invited mothers of in total 333 babies (including eight twins) were enrolled in the study (86.5%), of which half were recruited in the a-BF hospital (51.2%). A higher proportion of non-responders compared to responders were <25 years old, overweight/obese and had smoked during pregnancy. Except for primary caesarean section, birth mode and newborn characteristics were equally distributed. Responder and non-responder characteristics are presented in the Additional file 2.

**Fig. 2:**
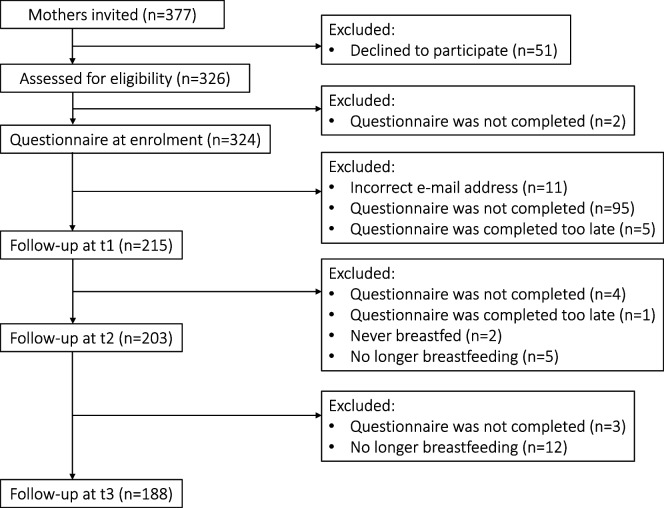
Participant flowchart. Legend: n= number of mothers, t0= first days postpartum, t1 | 2| 3= 2 | 6| 12 months postpartum, respectively

Participating mothers had completed study questionnaires at t1, t2 and t3 between 1.3–2.9, 5.2–8.2 and 11.2–14.2 months postpartum, respectively. Considering that mothers were only followed-up if ongoing breastfeeding was reported, the drop-out rate from t0 to t3 was 36.5% (Fig. 2).

### Breastfeeding duration and influencing factors

Almost all women for whom the information was available had initiated breastfeeding in the delivery room (94.7%, 286/302).

An exclusive breastfeeding duration of ≥4 months was reported by 157/205 (76.6%) mothers. The highest rate was reported among women who delivered in the a-BF hospital (83.6%, 92/110), followed by 76.5% (39/51) and 59.1% (26/44) by those recruited in the BF hospital and non-BF hospital, respectively. Overall, the median exclusive breastfeeding duration was 5.7 months. One year after birth, 65.7% of mothers reported ongoing breastfeeding; including 71.9% (82/114), 58.3% (28/48) and 57.8% (26/45) of mothers who delivered in the a-BF, BF and non-BF hospital, respectively (Table 1).

**Table 1:** Breastfeeding rates of first-time mothers during child's first year of life

	**2 months postpartum** (*N* = 215)	**6 months postpartum** (*N* = 210)	**12 months postpartum** (N = 207)
Exclusively breastfeeding [n (%)]	176 (81.9)	78 (37.1)	8 (3.9)
Partial breastfeeding [n (%)]	32 (14.9)	113 (53.8)	128 (61.8)
No longer breastfeeding [n (%)]	5 (2.3)	17 (8.1)	69 (33.3)
Never breastfed [n (%)]	2 (0.9)	2 (1.0)	2 (1.0)

Mothers who exclusively breastfed ≥ 4 months were less likely of having delivered by caesarean section. (32.5% vs. 43.8%), been obese before pregnancy (4.1% vs. 8.7%) and smoked during pregnancy (1.4% vs. 6.4%) (Table 2). Mothers who exclusively breastfed ≥ 4 months stated more often to be highly motivated to breastfeed shortly after delivery (96.8% vs. 87.2%) and felt more comfortable breastfeeding at t0 and t1 (27.1% vs. 14.9% and 55.6% vs. 19.0%) (Additional file 3).

**Table 2:** Maternal characteristics presented by breastfeeding duration and status

Variable	Exclusively breastfeeding					Ongoing breastfeeding 12 months postpartum				
	** < 4 months**		** ≥ 4 months**		*p*-value	**No**		**Yes**		*p*-value
	MD		MD			MD		MD		
**Maternal characteristics [n]**		**48**		**157**			**62**		**136**	
Maternal age at birth in years [median (range)]	0	34 (27–46)	0	34 (22–44)	0.188^t^	0	34 (22–46)	0	34 (25–44)	0.581^t^
BMI	2		11		0.400^t^	5		6		**0.007**^**t**^
< 18.5 kg/m^2^ [n (%)]		6 (13.0)		4 (2.7)			1 (1.8)		7 (5.4)	
18.5—24.9 kg/m^2^ [n (%)]		28 (60.9)		111 (76.0)			37 (64.9)		95 (73.1)	
25—29.9 kg/m^2^ [n (%)]		8 (17.4)		25 (17.1)			14 (24.6)		21 (16.2)	
≥ 30 kg/m^2^ [n (%)]		4 (8.7)		6 (4.1)			5 (8.8)		7 (5.4)	
Smoking [n (%)]	1	3 (6.4)	14	2 (1.4)	-^cx^	4	4 (6.9)	11	1 (0.8)	-^cx^
Alcohol [n (%)]	21	1 (3.7)	80	0 (0.0)	-^cx^	29	0 (0.0)	72	0 (0.0)	-
Migratory background [n (%)]	5	14 (32.6)	26	35 (26.7)	0.460^c^	11	9 (17.6)	17	38 (31.9)	0.056^c^
Subjective social status [median (range)]	6	4.00 (1–7)	24	4.00 (1–7)	0.510^t^	12	4.00 (1–7)	16	4.00 (1–7)	0.127^t^
Housing	4		23		-^cx^	10		15		-^cx^
Parents [n (%)]		39 (88.6)		127 (94.8)			50 (96.2)		114 (94.2)	
Mother (with partner) [n (%)]		5 (11.4)		6 (4.5)			2 (3.8)		7 (5.8)	
Father (with partner) [n (%)]		0 (0.0)		0 (0.0)			0 (0.0)		0 (0.0)	
Others [n (%)]		0 (0.0)		1 (0.7)			0 (0.0)		0 (0.0)	
**Birth characteristics [n]**		**48**		**157**			**62**		**136**	
Maternity clinic	0		0		**0.005**^**c**^	0		0		0.137^c^
Anthroposophic baby-friendly (a-BF) certified hospital [n (%)]		18 (37.5)		92 (58.6)			28 (45.2)		82 (60.3)	
Baby-friendly (BF) certified hospital [n (%)]		12 (25.0)		39 (24.8)			17 (27.4)		28 (20.6)	
Maximum care provider not certified as baby-friendly (non-BF) [n (%)]		18 (37.5)		26 (16.6)			17 (27.4)		26 (19.1)	
Twin births [n (%)]	0	7 (14.6)	0	0 (0.0)	-^cx^	0	6 (9.7)	0	0 (0.0)	-^cx^
Gestational age in weeks + days [median (range)]	0	39 + 6 (37 + 1–42 + 1)	0	40 + 1 (37 + 1–42 + 3)	**0.022**^**t**^	0	40 + 0 (37 + 1–42 + 0)	0	40 + 2 (37 + 2–42 + 3)	**0.027**^**t**^
Birth Mode	0		0		-^cx^	1		0		-^cx^
Vaginal [n (%)]		21 (43.8)		73 (46.5)			20 (32.8)		66 (48.5)	
Vaginal instrumental [n (%)]		6 (12.5)		33 (21.0)			16 (26.2)		27 (19.9)	
Scheduled caesarean section [n (%)]		11 (22.9)		9 (5.7)			12 (19.7)		8 (5.9)	
Unplanned caesarean section [n (%)]		9 (18.8)		40 (25.5)			12 (19.7)		34 (25.0)	
Emergency caesarean section [n (%)]		1 (2.1)		2 (1.3)			1 (1.6)		1 (0.7)	
PDA [n (%)]	0	35 (72.9)	0	102 (65.0)	0.306^c^	0	46 (75.2)	0	88 (64.7)	0.186^c^
Days in hospital [mean (standard deviation)]	13	3.57 (1.420)	49	2.87 (0.948)	**0.009**^**t**^	19	3.33 (1.267)	42	2.86 (0.967)	**0.036**^**t**^
**Newborn characteristics**^a^** [n]**		**54**		**157**			**67**		**136**	
Initiate breastfeeding in delivery room^a^ [n (%)]	3	46 (85.2)	12	137 (94.5)	-^cx^	4	58 (92.1)	11	120 (96.0)	-^cx^
Postpartum separation^a^[n (%)]	6	8 (16.7)	16	29 (20.6)	0.779^c^	9	9 (15.5)	16	21 (17.5)	0.893^c^
Sex^a^	1		0		-^cx^	0		0		-^cx^
Female [n (%)]		24 (45.3)		72 (45.9)			30 (44.8)		65 (47.8)	
Male [n (%)]		29 (54.7)		84 (53.5)			37 (55.2)		70 (51.5)	
Ambiguous [n (%)]		0 (0.0)		1 (0.6)			0 (0.0)		1 (0.7)	
Weight^a^	0		0		**0.023**^**t**^	0		0		**0.046**^**t**^
< 2500 g [n (%)]		4 (7.4)		1 (0.6)			3 (4.5)		0 (0.0)	
2500—4000 g [n (%)]		45 (83.3)		141 (89.8)			57 (85.1)		121 (89.0)	
> 4000 g [n (%)]		5 (9.3)		15 (9.6)			7 (10.4)		15 (11.0)	
Apgar 5'^a^ [median (range)]	0	10 (8–10)	0	10 (7–10)	0.648^t^	0	10 (8–10)	0	10 (7–10)	0.090^t^
Apgar 10'^a^ [median (range)]	0	10 (9–10)	0	10 (8–10)	0.733^t^	0	10 (8–10)	0	10 (8–10)	0.876^t^

MD: Missing Data (MD concerning exclusively breastfeeding: 121 regarding the number of mothers and 122 regarding the number of children, MD concerning breastfeeding 12 months postpartum: 128 regarding the number of mothers and 130 regarding the number of children)

*PDA* Peridural/epidural anaesthesia, *BMI* Body mass index, *Apgar* Apgar score includes appearance/pulse/grimace/activity/respiration of a newborn

^a^In terms of the number of children (otherwise in terms of the number of mothers)

^t^calculated by t-test

^c^calculated by chi-square-test

^cx^the chi-square-test cannot be performed due to the sample size with an expected cell frequency < 5

Multivariate analyses revealed that mothers who felt very comfortable with breastfeeding 2 months postpartum were 7 times more likely to breastfeed exclusively at least 4 months (Table 3).

**Table 3:** Multivariate analysis of factors supporting exclusive breastfeeding ≥ 4 months

	**Exclusive breastfeeding ≥ 4 months**		
	**aOR**	**CI 95%**	*p*-value
**Age of mother at birth**	1.01	(0.90, 1.13)	0.862
**Sex of the child**			
Female	1		
Male	0.74	(0.31, 1.75)	0.492
**Birth weight**	1.00	(1.00, 1.00)	0.213
**Birth Mode**			
Caesarean section	1		
Spontaneous delivery	1.16	(0.43, 3.12)	0.771
Vaginal-instrumental delivery	1.97	(0.48, 8.11)	0.345
**Maternity clinic**			
non-BF	1		
BF	1.27	(0.31, 5.29)	0.74
a-BF	1.59	(0.41, 6.14)	0.498
**Planned duration of exclusive breastfeeding**			
< 4 months	1		
≥ 4 until < 7 months	4.08	(0.29, 57.77)	0.299
≥ 7 months	2.71	(0.17, 42.84)	0.478
**Active offer of breastfeeding counselling in the hospital**			
No	1		
Yes	1.03	(0.29, 3.60)	0.964
**Support at the start of breastfeeding in hospital**			
Neither good nor not good/rather not got/not good at all	1		
Rather good	1.25	(0.38, 4.12)	0.713
Very good	1.39	(0.41, 4.70)	0.600
**Comfort concerning breastfeeding/feeding in the first days postpartum**			
Mediocre/rather not comfortable/not comfortable at all	1		
Rather comfortable	1.42	(0.53, 3.79)	0.489
Very comfortable	1.79	(0.49, 6.59)	0.381
**Comfort concerning breastfeeding/feeding 2 months postpartum**			
Mediocre/rather not comfortable/not comfortable at all	1		
Rather comfortable	**3.33**	**(1.20, 9.22)**	**0.021**
Very comfortable	**7.25**	**(2.11, 24.90)**	**0.002**

*aOR* Adjusted Odds Ratio, all variables presented in Table 3 were included in the model, irrespective of their statistical significance

*CI* Confidence interval

### Attitudes, perceptions and motivation towards breastfeeding over the first year of the child’s life

**Fig. 3:**
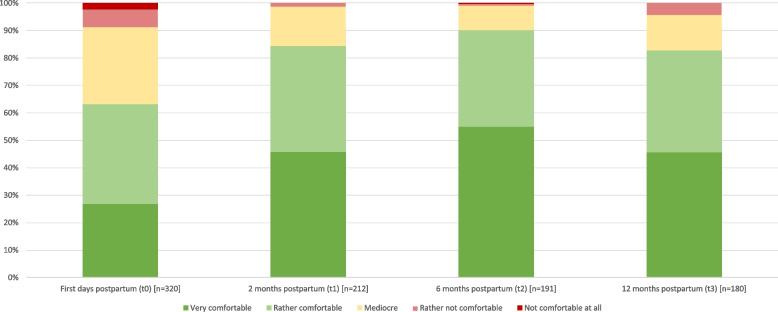
Maternal comfort with the breastfeeding/feeding situation over time. Legend: asymptotic significance calculated by two-factor variance analysis for ranks according to Friedman: t0-t1 0.000, t0-t2 0.000, t0-t3 0.007, t1-t2 1.000, t1-t3 1.000, t2-t3 0.215

Over the course of the first year, maternal comfort with the baby’s nutritional situation increased by 26.9% from birth to six months postpartum and then dropped by 7.3% until one year postpartum (Fig. 3). When considering the feeding modality, more mothers who exclusively breastfed felt very comfortable than those who were partially breastfeeding, no longer or never breastfeeding (t1: 52.0% vs. 16.7% vs. 20.0% vs. 0.0% | t2: 68.8% vs. 48.1% vs. 20.0%). However, 12 months postpartum, mothers who were no longer breastfeeding reported slightly more often high comfort with their baby’s nutritional situation compared to those who continued to breastfeed (56.9% vs. exclusively: 50.0% vs. partially: 40.5%).

Most breastfeeding mothers agreed/strongly agreed to perceive breastfeeding practical, with an increasing tendency during the child's first year of life (t1: 81.9%, t3: 92.1%), while their perception of breastfeeding being time consuming decreased over time (t1: 64.7%, t3: 30.1%). Yet, about half of the breastfeeding mothers agreed/strongly agreed that breastfeeding restricted their independency and that they found breastfeeding exhausting, with almost no change of these estimations over the course of the first year of their child’s life (t1: 61.3%, t3: 55.5%; t1: 41.2%, t3: 38.9%, respectively). Pain during breastfeeding and abstaining from alcohol and smoking played a minor role. (Additional file 4).

Regarding the reasons for breastfeeding, most breastfeeding mothers agreed/strongly agreed that they were breastfeeding because it is the natural form of nutrition for the child (t1: 97.5%, t3: 93.7%), they want to support the child's health (t1: 100.0%, t3: 98.4%), they can strengthen the bond with their child in this way (t1: 87.8%, t3: 93.0%) and because of the good rhythm between child and mother (t1: 68.8%, t3: 85.8%). Supporting their own health and the cost advantage played a minor role; yet, around half of the breastfeeding mothers agreed/strongly agreed with these statements (t1: 55.9%, t3: 54.0%; t1: 46.8%, t3: 46.1%, respectively). (Additional file 5).

### Breastfeeding counselling

**Fig. 4:**
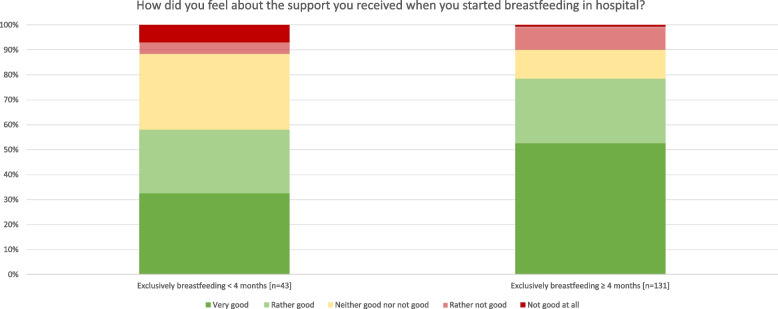
Maternal perception of breastfeeding counselling in the hospital. Legend: question answered by mothers 2 months postpartum. = 0.01 (calculated by Mann–Whitney U-test)

More mothers who exclusively breastfed ≥ 4 months reported feeling (very) good about the support in the hospital when initiating breastfeeding compared to those exclusively breastfeeding < 4 months (Fig. 4). In general, mothers who gave birth in the a-BF hospital stated more often that breastfeeding/feeding was sufficiently discussed with them in the hospital (92.6%), compared to the BF (77%) and non-BF (65.9%) hospital and reported active breastfeeding counselling more frequently (a-BF 89.5% vs. BF 84.7% vs. non-BF 63.5%).

Mothers who exclusively breastfed ≥ 4 months were satisfied with the breastfeeding counselling in hospital to higher rates than mothers who breastfed < 4 months because of the professionality (74.4% vs. 56.9%), consideration of the individual needs (66.4% vs. 34.9%), addressing individual problems (74.3% vs. 58.2%), understanding the individual needs of the child (62.6% vs. 48.9%), realisability of what has been communicated (76.7% vs. 46.5%), strengthening the mothers confidence with breastfeeding (68.2% vs. 38.1%) and making them feeling competent (53.4% vs. 32.6%), as well as involvement of the partner (38.3% vs. 35.0%).

Before and during the hospital stay, distribution of advisory sources concerning breastfeeding/feeding did not differ between mothers who breastfed ≥ 4 months and those who breastfed shorter, however mothers who breastfed < 4 months reported twice as often support by a lactation counselor (18.2% vs. 9.0%). Most mothers stated that they would find breastfeeding counselling particularly helpful before and shortly after birth, especially those who exclusively breastfed 4 months (Additional file 6).

## Discussion

In our study cohort, approximately three quarters of participating mothers exclusively breastfed 4 months or more; almost two thirds reported ongoing breastfeeding at 12 months. Generally, the exclusive breastfeeding rates recorded in this study, conducted between November 2022 and May 2024, were higher than those observed in Germany in 2018/2019 (exclusively breastfeeding 4 months postpartum: 77% vs. 57%, partial breastfeeding 12 months postpartum: 62% vs. 41%; [17]). This trend of increasing breastfeeding rates over time was also seen in previous studies [17]. Factors influencing the exclusive breastfeeding duration include maternal age, pre-pregnancy BMI, mode of delivery, occupation and income, breastfeeding support and delivery in a baby-friendly hospital [25, 26], as well as newborn characteristics such as birth weight and sex [7, 10]. Equally, in our study, women who planned to exclusively breastfeed for 4 to 7 months, who gave birth spontaneously and in a baby-friendly certified hospital, with a norm-value pre-pregnancy BMI, with higher assessment of their socio-economic status and who did not smoke during pregnancy were more likely to exclusively breastfeed ≥ 4 months. We showed that exclusive breastfeeding for ≥ 4 months was most strongly associated with maternal comfort regarding the breastfeeding/feeding situation 2 months postpartum; this has not been described in previous studies. Overall, maternal comfort with the breastfeeding situation increased in the first 6 months after birth, which can be reassuring information for mothers, especially those experiencing initial breastfeeding challenges. Furthermore mothers who exclusively breastfed ≥ 4 months reported higher satisfaction with the breastfeeding counselling in hospital than mothers who breastfed < 4 months because of the professionality, consideration of the individual needs, addressing individual problems, understanding the individual needs of the child, realisability of what has been communicated, strengthening the mothers confidence with breastfeeding and making them feeling competent and with involvement of the partner.

Most mothers elect to breastfeed before giving birth [27]. Aside from breastfeeding being considered practical, reasons for breastfeeding were a natural form of nutrition, strengthening the mother–child bond and supporting the baby's health; the latter consistent with results from a previous study [26]. Overtime, potential strengthening of the mother–child bond as well as the good rhythm between both further increased as a reason to breastfeed. In our cohort almost all mothers stated that they wanted to breastfeed their child shortly after delivery and only 4.9% pursued an exclusive breastfeeding duration for < 4 months. However, the rate of mothers who eventually breastfed < 4 months was almost 25%, potentially related to the fact that the breastfeeding recommendation of exclusively 4–6 months was given in the study information. It cannot be ruled out that they might have felt obliged to state a longer period at inclusion/while being on the postnatal ward.

Antepartum breastfeeding education was shown to be an important factor for exclusive breastfeeding duration [28] and was also stated as helpful by mothers in our study. Two thirds found breastfeeding counselling especially helpful directly after birth, with more mothers who breastfed ≥ 4 months having felt more supported in the hospital when initiating breastfeeding. While in the present study, the influence of the maternity hospitals on the exclusive breastfeeding duration was not significant, mothers who gave birth in the a-BF hospital and in the BF hospital were 1.6 times and 1.3 times more likely to exclusively breastfed ≥ 4 months than in the non-BF hospital, respectively. This trend was also shown with regard to ongoing breastfeeding one year postpartum. Mothers who gave birth in the a-BF hospital assessed the support received more positively than those recruited in the BF hospital and even more than in the non-BF hospital. Mothers who gave birth in the a-BF hospital stated more frequently that all their questions were answered sufficiently, and breastfeeding counselling was actively offered than mothers who delivered in the BF and non-BF hospitals. This stresses the importance of the individualized approach when accompanying mothers, fathers and their newborns after birth. Our results can be interpreted as an indication that the different concepts of the hospitals including the implementation and quality of breastfeeding counselling could be relevant regarding the breastfeeding duration.

For the long-term sustainability of breastfeeding, a support from the general community is particularly important [12]. Most mothers in our study desired ongoing breastfeeding counselling following discharge. Most commonly mothers received follow-up breastfeeding support by a midwife, with rates similarly high as in previous studies [29]. Only 18% of the mothers reported support from a specialized breastfeeding counsellor (international board of lactation consultant examiner, IBCLC). While this service seems underused considering the remaining high rates of short breastfeeding durations, it is not covered by insurance in Germany and therefore is not equally accessible for all mothers in need. An easy-to-use questionnaire can be used to identify mothers at risk [30] before discharge, as well as during follow-up appointments with the midwife, paediatrician or gynaecologist. Continued professional counselling is suggested to support realistic expectations, positive coping, self-efficacy and mobilization of social support [31] to reduce premature breastfeeding cessation.

### Limitations

Limitations were identified in the study. It is conceivable that mothers who were open to breastfeeding and tended to have a more positive breastfeeding attitude and confidence participated in this study. In this concern, the questionnaires were formulated as neutrally as possible regarding the attitude and breastfeeding success. The questionnaires were developed specifically for the study due to the lack of previous research. They were tested before the start of the study, but have not been validated. No further questionnaires were sent to mothers who were no longer breastfeeding, which was decided on the basis of the primary outcome regarding the duration of breastfeeding and to reduce study burden in first time mothers with young infants. Nevertheless, this and the potential self-selection may limit generalisation and comparability with the general population. Mothers with risk factors were included in the study, but these were less represented, such as mothers who were young (aged < 25 years), obese or smoked. In contrast to many other studies, mothers with twins were not excluded. Equally, mode of delivery, including emergency deliveries, were considered, while pre-existing severe or chronic health conditions were not but which may influence the outcome of breastfeeding initiation, success and duration. It should be noted that this is an observational study. Findings regarding the influence of different hospital concepts are addressed in more depth in the qualitative interviews of this study, results will be reported separately.

## Conclusion

Our results emphasize the pivotal role of the mother’s comfort in the breastfeeding process. Maternal comfort with the breastfeeding situation 2 months postpartum is associated with a longer exclusive breastfeeding duration, indicating the importance of ensuring maternal comfort with breastfeeding in the care of expectant and new mothers also beyond the hospital setting. Key aspects of maternal comfort need to be further explored. In-depth inside on how maternal comfort is shaped before having given birth, in the hospital setting and over-time following discharge were collected in complementing qualitative interviews which will be presented in future, also specifically addressing the specific breastfeeding management and support received in the different types of hospitals. This will enlarge the view of breastfeeding promotion and identify parent-centred strategies for sustainable breastfeeding.

## Supplementary Information

Additional file 1: Overview of study questionnaire.

Additional file 2: Characteristics of participants and non-responders.

Additional file 3: Mother’s breastfeeding attitudes and perceptions by exclusively breastfeeding 4 months and breastfeeding 12 months postpartum.

Additional file 4: Sensation of breastfeeding by nursing mothers over time. Legend: t1 | 2| 3 = 2 | 6| 12 months postpartum asymptotic significance calculated by two-factor variance analysis for ranks according to Friedman: pleasant (t1-t2 0.046, t1-t3 0.802, t2-t3 0.563), enjoyable (t1-t2 1.000, t1-t3 0.802, t2-t3 1.000), practical (t1-t2 0.758, t1-t3 0.529, t2-t3 1.000), time consuming (t1-t2 0.000, t1-t3 0.000, t2-t3 0.847), restricts independence (t1-t2 1.000, t1-t3 1.000, t2-t3 1.000), exhausting (t1-t2 1.000, t1-t3 0.716, t2-t3 0.802), painful (t1-t2 0.002, t1-t3 0.025, t2-t3 1.000), difficult to give up alcohol/smoking (t1-t2 0.381, t1-t3 0.716, t2-t3 1.000).

Additional file 5: Nursing mothers’ reasons for breastfeeding over time. Legend: t1 | 2| 3 = 2 | 6| 12 months postpartum asymptotic significance calculated by two-factor variance analysis for ranks according to Friedman: natural nutrition (t1-t2 1.000, t1-t3 0.040, t2-t3 0.092), strengthening the bond (t1-t2 1.000, t1-t3 1.000, t2-t3 1.000), supporting child’s health (t1-t2 1.000, t1-t3 0.442, t2-t3 0.765), supporting mother’s health (t1-t2 1.000, t1-t3 0.902, t2-t3 0.442), practical (t1-t2 1.000, t1-t3 1.000, t2-t3 1.000), cheaper (t1-t2 0.951, t1-t3 1.000, t2-t3 0.173), good rhythm between child and mother (t1-t2 0.004, t1-t3 0.043, t2-t3 1.000).

Additional file 6: Mothers'assessments of when breastfeeding counselling would be particularly helpful by exclusively breastfeeding 4 months postpartum. Legend: U2: paediatric examination between 3rd and 10th day of life, U3: in the 4th to 5th week of life, U4: between the 3rd and 4th month of life, U5: between the 6th and 7th month of life. *p*-value calculated by chi-square-test regarding exclusive breastfeeding ≥ vs. <4 months: registration appointment in clinic 0.067, birth preparation course 0.075, directly after birth 0.510, on the day of birth 0.011, U2 0.425, first days at home 0.001; test cannot be performed due to the sample size with an expected cell frequency < 5 for start of pregnancy|admission to the clinic|U3|in the second month postpartum|U4|U5.

## Data Availability

Due to participant confidentiality and legal restrictions the datasets generated during the study are not publicly available. However, limited data may be available from the corresponding author on reasonable request and with appropriate approvals.

## References

[1] Chowdhury R, Sinha B, Sankar MJ, Taneja S, Bhandari N, Rollins N, et al. Breastfeeding and maternal health outcomes: a systematic review and meta-analysis. Acta Paediatr. 2015;104(467):96–113. 26172878 10.1111/apa.13102 PMC4670483

[2] Stillen in Deutschland: Kontext und Herausforderungen [Breastfeeding in Germany: context and challenges] [https://www.bmel.de/SharedDocs/Downloads/DE/Broschueren/nationale-stillstrategie.pdf?__blob=publicationFile&v=11]

[3] Pattison KL, Kraschnewski JL, Lehman E, Savage JS, Downs DS, Leonard KS, et al. Breastfeeding initiation and duration and child health outcomes in the first baby study. Prev Med. 2019;118:1–6. 30287329 10.1016/j.ypmed.2018.09.020 PMC6322935

[4] Horta BL. Loret de Mola C, Victora CG: Long-term consequences of breastfeeding on cholesterol, obesity, systolic blood pressure and type 2 diabetes: a systematic review and meta-analysis. Acta Paediatr. 2015;104(467):30–7. 26192560 10.1111/apa.13133

[5] Breastfeeding: recommendations [https://www.who.int/health-topics/breastfeeding#tab=tab_2]

[6] Nationale Stillkommission: Weiterhin 4 bis 6 Monate ausschließlich stillen [National Breastfeeding Commission: continuing to breastfeed exclusively for 4 to 6 months] [https://www.bfr.bund.de/de/presseinformation/2015/12/nationale_stillkommission__weiterhin_4_bis_6_monate_ausschliesslich_stillen-194091.html]

[7] Tracz J, Gajewska D. Factors influencing the duration of breastfeeding among polish women. J Mother Child. 2020;24(1):39–46. 33074176 10.34763/jmotherandchild.2020241.2006.000007 PMC8518111

[8] Lutsiv O, Pullenayegum E, Foster G, Vera C, Giglia L, Chapman B, et al. Women’s intentions to breastfeed: a population-based cohort study. BJOG. 2013;120(12):1490–8. 23859024 10.1111/1471-0528.12376

[9] Gutierrez-de-Terán-Moreno G, Ruiz-Litago F, Ariz U, Fernández-Atutxa A, Mulas-Martín MJ, Benito-Fernández E, et al. Successful breastfeeding among women with intention to breastfeed: from physiology to socio-cultural factors. Early Hum Dev. 2022;164: 105518. 34864612 10.1016/j.earlhumdev.2021.105518

[10] Pande H, Unwin C, Håheim LL. Factors associated with the duration of breastfeeding: analysis of the primary and secondary responders to a self-completed questionnaire. Acta Paediatr. 1997;86(2):173–7. 9055888 10.1111/j.1651-2227.1997.tb08862.x

[11] Implementation of the baby-friendly hospital initiative [https://www.who.int/tools/elena/bbc/implementation-bfhi]

[12] Pérez-Escamilla R, Martinez JL, Segura-Pérez S. Impact of the baby-friendly hospital initiative on breastfeeding and child health outcomes: a systematic review. Matern Child Nutr. 2016;12(3):402–17. 26924775 10.1111/mcn.12294 PMC6860129

[13] Merten S, Dratva J, Ackermann-Liebrich U. Do baby-friendly hospitals influence breastfeeding duration on a national level? Pediatrics. 2005;116(5):e702-708. 16263985 10.1542/peds.2005-0537

[14] Introduction: anthroposophic medicine [https://www.ivaa.info/anthroposophic-medicine/introduction/]

[15] Ihre Geburt in Havelhöhe [Your birth at Havelhöhe] [https://www.havelhoehe.de/de/abteilungen-zentren/frauenheilkunde-und-geburtshilfe/geburtshilfe/ihre-geburt-in-havelhoehe/]

[16] Brettschneider AK, von der Lippe E, Lange C. Breastfeeding behaviour in Germany-News from KiGGS Wave 2. Bundesgesundheitsblatt Gesundheitsforschung Gesundheitsschutz. 2018;61(8):920–5. 29934682 10.1007/s00103-018-2770-7

[17] Hockamp N, Burak C, Sievers E, Rudloff S, Burmann A, Thinnes M, et al. Breast-feeding promotion in hospitals and prospective breast-feeding rates during the first year of life in two national surveys 1997–1998 and 2017–2019 in Germany. Public Health Nutr. 2021;24(9):2411–23. 33722333 10.1017/S1368980021001099 PMC10195543

[18] Information für Einweisende [Information for referrers] [https://neonatologie.charite.de/leistungen/information_fuer_einweisende#:~:text=Zusammen%20mit%20der%20Klinik%20für,bei%20uns%20behandelt%20werden%20können.]

[19] Klinik für Kinder- und Jugendmedizin - Neonatologie Westend [Clinic for paediatrics and youth medicine - neonatology Westend] [https://www.drk-kliniken-berlin.de/neonatologie-westend]

[20] Kuckartz U: Designs für die Mixed-Methods-Forschung [Designs for mixed-methods research]. In: Mixed Methods: Methodologie, Forschungsdesigns und Analyseverfahren. edn. Edited by Kuckartz U. Wiesbaden: Springer Fachmedien Wiesbaden; 2014: 57–98.

[21] Einheitliche Terminologie zur Säuglingsernährung. Aktualisierte Empfehlung (2007) der Nationalen Stillkommission von 1999 [Standardised terminology for infant feeding. Updated recommendation (2007) of the National Breastfeeding Commission of 1999 ] [https://www.mri.bund.de/fileadmin/MRI/Themen/Stillkommission/einheitliche_terminologie_zur_saeuglingsernaehrung.pdf]

[22] Hoebel J, Müters S, Kuntz B, Lange C, Lampert T. Messung des subjektiven sozialen Status in der Gesundheitsforschung mit einer deutschen Version der MacArthur Scale [Measuring subjective social status in health research with a German version of the MacArthur scale]. Bundesgesundheitsbl. 2015;58:749–57. 10.1007/s00103-015-2166-x. 10.1007/s00103-015-2166-x 25986532

[23] Migrationshintergrund [Migration background] [https://www.destatis.de/DE/Themen/Gesellschaft-Umwelt/Bevoelkerung/Migration-Integration/Glossar/migrationshintergrund.html]

[24] Hobbs AJ, Mannion CA, McDonald SW, Brockway M, Tough SC. The impact of caesarean section on breastfeeding initiation, duration and difficulties in the first four months postpartum. BMC Pregnancy Childbirth. 2016;16:90. 27118118 10.1186/s12884-016-0876-1 PMC4847344

[25] Asimaki E, Dagla M, Sarantaki A, Iliadou M. Main biopsychosocial factors influencing breastfeeding: a systematic review. Maedica (Bucur). 2022;17(4):955–62. 36818247 10.26574/maedica.2022.17.4.955 PMC9923068

[26] Bürger B, Schindler K, Tripolt T, Griesbacher A, Stüger HP, Wagner KH et al.: Factors associated with (exclusive) breastfeeding duration-results of the SUKIE-study. Nutrients 2022, 14(9). 10.3390/nu14091704 PMC9102851 35565672

[27] Ballesta-Castillejos A, Gómez-Salgado J, Rodríguez-Almagro J, Ortiz-Esquinas I, Hernández-Martínez A. Factors that influence mothers’ prenatal decision to breastfeed in Spain. Int Breastfeed J. 2020;15:97. 33203421 10.1186/s13006-020-00341-5 PMC7672988

[28] Yılmaz E, Doğa Öcal F, Vural Yılmaz Z, Ceyhan M, Kara OF, Küçüközkan T. Early initiation and exclusive breastfeeding: factors influencing the attitudes of mothers who gave birth in a baby-friendly hospital. Turk J Obstet Gynecol. 2017;14(1):1–9. 28913128 10.4274/tjod.90018 PMC5558311

[29] Kersting M, Hockamp N, Burak C et al.: Studie zur Erhebung von Daten zum Stillen und zur Säuglingsernährung in Deutschland – SuSe II [Study to collect data on breastfeeding and infant feeding in Germany - SuSe II]. In: 14 DGE-Ernährungsbericht Vorveröffentlichung Kapitel 3. Bonn (2020) Volume V1-V 34; https://www.dge.de/14-dge-eb/vvoe/kap3.

[30] Kronborg H, Vaeth M. Validation of the breastfeeding score-a simple screening tool to predict breastfeeding duration. Nutrients. 2019;11(12):2852. 31766388 10.3390/nu11122852 PMC6950692

[31] Fahey JO, Shenassa E. Understanding and meeting the needs of women in the postpartum period: the perinatal maternal health promotion model. J Midwifery Womens Health. 2013;58(6):613–21. 24320095 10.1111/jmwh.12139

