# Supplementary material for: How can we support the individual breastfeeding experience? Quantitative results from a mixed-methods study
Source: Int Breastfeed J. 2025 May 17;20:38. doi: 10.1186/s13006-025-00726-4 (PMC12085814; doi:10.1186/s13006-025-00726-4)
Supplement: Supplementary file 1 — Additional file 1: Overview of study questionnaire. [file 13006_2025_726_MOESM1_ESM.docx]

| **Additional file 1** Questions from the study questionnaires | | | | |
| --- | --- | --- | --- | --- |
| **No.** | **Question** | **Answer options** | **Type** | **Included in questionnaire**  Q1 at t0  Q2 at t1  Q3 at t2  Q4 at t3 |
| Breastfeeding motivation | | | | |
| 1 | I want to breastfeed my child. | 1 = Strongly disagree  2 = Disagree  3 = Neither agree nor disagree 4 = Agree  5 = Strongly agree | Likert scale  (1-5) | Q1 |
| 2 | I plan to exclusively breastfeed my child [number] months. | Free text | Open response | Q1 |
| Breastfeeding / feeding situation | | | | |
| 3 | How are you currently feeding your child? | 1. I exclusively breastfeed with breastmilk. 2. I breastfeed and supplement with fluids/formula/complementary food. 3. I stopped breastfeeding since [date]. 4. I have never breastfed. | Single choice | Q2; Q3; Q4 |
| 4 | Currently I breastfeed [number] times within 24 hours | Free text | Open response | Q2; Q3; Q4 |
| 5 | I breastfed exclusively until [date]. | Free text | Open response | Q2; Q3; Q4 |
| 6 | From the decision to stop breastfeeding until I successfully stopped, it took ___ days. | Free text | Open response | Q2; Q3; Q4 |
| 7 | How satisfied are you with the decision not to breastfeed any longer? | 1 = Not satisfied at all  2 = Rather not satisfied  3 = Mediocre 4 = Rather satisfied  5 = Very satisfied | Likert scale  (1-5) | Q2; Q3; Q4 |
| 8 | My child drinks or eats in addition to breastmilk...   1. ... liquids: [water/ tea/ sugar solution] since [date] (currently [amount] ml per day). 2. ... formula: [pre-nutrition] since [date] (currently [amount] ml per day). 3. ... complementary food: [vegetable porridge] since [date] (currently [number] meals per day). | Free text | Open response | Q2; Q3; Q4 |
| 9 | What was helpful for your process of stopping breastfeeding? | Free text | Open response | Q2; Q3; Q4 |
| 10 | Which breastfeeding aids have you tried before stopping? | 1. None 2. Create a cosy breastfeeding atmosphere 3. Eye contact with my child 4. Lactation tea 5. Supplemental nursing system 6. Lanolin/wool wax 7. ‘Donut’ 8. Breast caps 9. Nipple shaper 10. Breast pump 11. Other: [___] | Multiple choice | Q2; Q3; Q4 |
| 11 | How did you feel about introducing breastfeeding aids? | Free text | Open response | Q2; Q3; Q4 |
| 12 | How long did you try to continue breastfeeding with the help of breastfeeding aids? | [Number] weeks | Open response | Q2; Q3; Q4 |
| Breastfeeding perception and motivation | | | | |
| 13 | How comfortable do you currently feel with breastfeeding/feeding your child? | 1 = Not comfortable at all  2 = Rather not comfortable  3 = Mediocre 4 = Rather comfortable  5 = Very comfortable | Likert scale  (1-5) | Q1; Q2; Q3; Q4 |
| 14 | I breastfeed/breastfed because...  ... it is the natural form of nutrition for an infant.  ... I can strengthen a loving bond with my child.  ... I want to support my child's health (reduce allergies, strengthen the immune system, build up intestinal flora, ...).  ... it is good for me (health-wise, psychologically).  ... it is cheaper than the additional buying of baby food.  ... it is practical.  ... my child and I have found a good rhythm together. | 1 = Strongly disagree  2 = Disagree  3 = Neither agree nor disagree 4 = Agree  5 = Strongly agree | Likert scale  (1-5) | Q2; Q3; Q4 |
| 15 | To what extent do you agree with the following statements?  Breastfeeding …   1. is painful. 2. is pleasant. 3. is exhausting. 4. I can enjoy it to the full. 5. takes up a lot of time. 6. is practical. 7. restricts my independence. 8. I find it difficult to give up my enjoyment of alcoholic beverages, smoking etc. | 1 = Strongly disagree  2 = Disagree  3 = Neither agree nor disagree 4 = Agree  5 = Strongly agree | Likert scale  (1-5) | Q2; Q3; Q4 |
| 16 | I stopped breastfeeding / I am not breastfeeding because...   1. it is painful. 2. I lack the energy to do so. 3. breastfeeding due to the diagnosis [free text] of my child/me is not possible. 4. I am not allowed to breastfeed due to my medication. 5. I want to enable other family members (e.g. my partner) to feed my child. 6. I want to be independent of my child (e.g. at work). 7. I had to start working. 8. I want to maintain my breast shape. 9. I have had bad experiences (e.g. mastitis/breast inflammation). 10. I have experienced traumatization/abuse and the the closeness is difficult for me to bear. 11. Other… | 1 = Strongly disagree  2 = Disagree  3 = Neither agree nor disagree 4 = Agree  5 = Strongly agree | Likert scale  (1-5) | Q2; Q3; Q4 |
| 17 | To what extend do you agree with the following statements? I assume that breastfeeding ...   1. ... is painful. 2. ... is pleasant. 3. ... is strength-sapping. 4. ... would be fully enjoyable for me 5. ... takes a lot of time. 6. ... is practical. 7. ... limits my independence. 8. ... prevents my enjoyment of alcoholic   drinks, smoking, etc.   1. Others… | 1 = Strongly disagree  2 = Disagree  3 = Neither agree nor disagree 4 = Agree  5 = Strongly agree | Likert scale  (1-5) | Q2 |
| Maternity clinic and breastfeeding counselling | | | | |
| 18 | What were the decisive reasons for your choice of maternity clinic?  It was important to me that...   1. ... that I was treated individually. 2. ... I was given a break from everyday life. 3. ... I can feel safe and secure. 4. ... I like the rooms and surroundings. 5. ... the maternity clinic is certified as baby-friendly. 6. ... the maximum possible medical care is available on site. 7. ... I know the staff there. 8. ... it was personally recommended to me. 9. ... it is close to where I live. 10. Others: [___] | 1 = Strongly disagree  2 = Disagree  3 = Neither agree nor disagree 4 = Agree  5 = Strongly agree | Likert scale  (1-5) | Q2 |
| 19 | Was breastfeeding/feeding addressed with you in hospital? | 1. Yes, and I had no more questions. 2. Yes, but my questions were not answered sufficiently. 3. No, but I would have liked to talk about it. 4. No, but I didn't feel the need to talk about it. | Single choice | Q1 |
| 20 | What applies to your time in hospital? | 1. I was actively offered advice on breastfeeding/feeding my child. 2. I asked questions about breastfeeding/feeding my child myself. 3. There was no opportunity to discuss questions about breastfeeding/feeding. | Multiple choice | Q1 |
| 21 | How did you find the support at the beginning of breastfeeding? | 1 = Not good at all  2 = Rather not good  3 = Neither good nor not good  4 = Rather good  5 = Very good | Likert scale  (1-5) | Q2 |
| 22 | I was given advice on breastfeeding/feeding by... | 1. No support received 2. Family 3. Friends 4. Familiar person 5. Flyer/ brochure 6. Internet research 7. Midwife during the antenatal period 8. Gynaecologist during the antenatal appointments 9. Midwife/ nurse in the delivery room 10. Midwife/ nurse in the postnatal ward 11. Lactation consultant on the postnatal ward 12. Doctor on the postnatal ward (e.g. gynaecologist, paediatrician) 13. Other: [___] | Multiple choice | Q1 |
| 23 | Who provided support with breastfeeding/feeding after leaving the hospital? | 1. No support received 2. Doctor (e.g. paediatrician, gynaecologist) 3. Midwife at home 4. Lactation consultant 5. Family 6. Friends 7. Familiar person 8. Flyer/ brochure 9. Internet research 10. Other: [___] | Multiple choice | Q2 |
| 24 | At what point would a conversation about breastfeeding be particularly helpful for you? | 1. At the start of pregnancy 2. At the registration appointment in the clinic 3. At the antenatal classes 4. On admission to the clinic 5. Directly after the birth 6. On the day of birth 7. At U2 (3rd to 10th day of life) 8. During the first few days at home 9. At U3 (4th to 5th week of life) 10. During the 2nd month of life 11. For U4 (3rd to 4th month of life) 12. At U5 (6th to 7th month of life) 13. At any other time: [___] | Multiple choice | Q2 |
| 25 | I was satisfied with the advice on breastfeeding in the hospital because...   1. it was professional and good. 2. I was able to implement what I was told directly. 3. it made me feel competent. 4. it strengthened my confidence in breastfeeding. 5. my individual needs were considered. 6. individual problems were addressed. 7. I was supported in understanding the individual needs of my child. 8. my partner was involved. 9. Others: [___] | 1 = Strongly disagree  2 = Disagree  3 = Neither agree nor disagree 4 = Agree  5 = Strongly agree | Likert scale  (1-5) | Q2 |
| 26 | Looking back, what was particularly important to you during the breastfeeding counseling? | Free text | Open response | Q2 |
| 27 | I was not satisfied with the advice on breastfeeding in the hospital because...   1. I lacked information. 2. I could not implement what I was told. 3. I felt taken by surprise (the timing was not appropriate). 4. my privacy was not respected. 5. I felt inadequate as a result. 6. my needs were not taken into account. 7. individual problems were not given sufficient attention. 8. my partner was not involved. 9. Others: [___] | 1 = Strongly disagree  2 = Disagree  3 = Neither agree nor disagree 4 = Agree  5 = Strongly agree | Likert scale  (1-5) | Q2 |
| 28 | Looking back, what would you wish had been different in terms of breastfeeding advice? | Free text | Open response | Q2 |
| Partners Involvement | | | | |
| 29 | Are you currently in a relationship? | a) Yes  b) No | Single choice | Q2; Q3; Q4 |
| 30 | What is your partner's attitude towards breastfeeding?  He/she... | 1. thinks breastfeeding is good and important. 2. has no opinion about it. 3. did not want me to breastfeed. 4. I do not know. | Single choice | Q2 |
| 31 | What role does your partner play in breastfeeding/feeding? | 1. No role 2. is attentive to my needs (e.g. brings me a glass of water when I am thirsty). 3. uses other moments outside of breastfeeding/feeding to establish close contact with our child. 4. supports me emotionally (e.g. by being present and encouraging me in what I am doing). 5. makes sure that - in addition to being parents - we nurture our partnership (e.g. by compliments). 6. takes responsibility for decisions that affect us as a family. 7. Other: [___] | Single choice | Q2; Q3; Q4 |
| 32 | How satisfied are you with your partner's involvement with regard to breastfeeding/feeding? | 1 = Not satisfied at all  2 = Rather not satisfied  3 = Mediocre 4 = Rather satisfied  5 = Very satisfied | Likert scale  (1-5) | Q2; Q3; Q4 |
| 33 | How could your partner support you in the success of breastfeeding/feeding your child? | Free text | Open response | Q2; Q3; Q4 |
| Socio-demographics | | | | |
| 34 | My partner…  … is [___] years old.  … has [___] children with a different partner. | Free text | Open response | Q2 |
| 35 | Do you plan to start working? | a) Yes  b) No | Single choice | Q2 |
| 36 | From when do you plan to work and for how many hours? | Free text | Open response | Q2 |
| 37 | Are you currently working? | a) Yes  b) No | Single choice | Q3; Q4 |
| 38 | I currently work ___ hours per month. | Free text | Open response | Q3; Q4 |
| 39 | Which of the following information on employment currently applies to  a) you and  b) your partner? | 1. Temporary leave of absence (e.g. parental leave) 2. Not employed (incl. students) 3. Part-time/hourly employed 4. Full-time employed 5. In training 6. Unemployed | Single choice | Q2 |
| 40 | Who does your child mainly live with? | a) Physical parents  b) Mother (with partner)  c) Father (with partner)  d) Other | Single choice | Q2 |
| 41 | In which country were you as the mother, your partner and the child's grandparents born? | a) In Germany  b) In another country | Single choice | Q2 |
| 42 | Where would you place yourself on the social ladder?  *1 = least money, lowest education, worst jobs*  *10 = most money, highest education, best jobs* | 1 = Lowest social status 10 = Highest social status | Likert scale  (1-10) | Q2 |
| All questions were freely translated by the authors. | | | | |
