# Supplementary material for: How can we support the individual breastfeeding experience? Quantitative results from a mixed-methods study
Source: Int Breastfeed J. 2025 May 17;20:38. doi: 10.1186/s13006-025-00726-4 (PMC12085814; doi:10.1186/s13006-025-00726-4)
Supplement: Supplementary file 2 — Additional file 2: Characteristics of participants and non-responders. [file 13006_2025_726_MOESM2_ESM.docx]

| **Additional file 2** Characteristics of participants and non-responders | | | |
| --- | --- | --- | --- |
|  | **MD (Responder\|Nonresponder)** | **Responder** | **Nonresponder** |
| Maternal characteristics [n] |  | 326 | 51 |
| Maternal age at birth in years [median (range)] | 0\|1 | 33 (19-46) | 32 (18-44) |
| 18 - 24 years [n (%)] |  | 14 (4.3) | 7 (14.0) |
| 25 - 35 years [n (%)] |  | 214 (65.6) | 29 (58.0) |
| ≥ 36 years [n (%)] |  | 98 (30.1) | 14 (28.0) |
| BMI [n] | 20\|12 | 306 | 39 |
| <18.5 kg/m^2^ |  | 15 (4.9) | 3 (7.7) |
| 18.5 - 24.9 kg/m^2^ |  | 214 (69.9) | 20 (51.3) |
| 25 - 29.9 kg/m^2^ |  | 57 (18.6) | 11 (28.2) |
| ≥ 30 kg/m^2^ |  | 20 (6.5) | 5 (12.8) |
| Smoking [n/N (%)] | 25\|4 | 10/301 (3.3) | 4/47 (8.5) |
| Alcohol [n/N (%)] | 154\|17 | 2/172 (0.6) | 0/34 (0.0) |
| Migratory background [n/N (%)] | 117\|- | 63/209 (30.1) | - |
| Educational and financial situation [median (range)] | 118\|- | 4.00 (1-7) | - |
| Housing [n] | 113\|- | 213 | - |
| Parents [n (%)] |  | 199 (93.4) | - |
| Mother (with partner) [n (%)] |  | 13 (6.1) | - |
| Father (with partner) [n (%)] |  | 0 (0.0) | - |
| Others [n (%)] |  | 1 (0.5) | - |
| Birth characteristics [n] |  | 326 | 51 |
| Maternity clinic [n] | 0\|0 | 326 | 51 |
| Anthroposophic clinic certified as baby-friendly [n (%)] |  | 167 (51.2) | 17 (33.3) |
| Clinic certified as baby-friendly [n (%)] |  | 74 (22.7) | 8 (15.7) |
| University hospital not certified as baby-friendly [n (%)] |  | 85 (26.1) | 26 (51.0) |
| Twin birth [n (%)] | 0\|1 | 8 (2.5) | 0 (0.0) |
| Gestational age in weeks+days [median (range)] | 0\|0 | 40+1 (37+1-42+3) | 39+3 (37+0-42+0) |
| Birth Mode [n] | 2\|1 | 324 | 50 |
| Vaginal [n (%)] |  | 145 (44.8) | 24 (48.0) |
| Vaginal instrumental [n (%)] |  | 63 (19.4) | 9 (18.0) |
| Scheduled caesarean section [n (%)] |  | 26 (8.0) | 2 (4.0) |
| Unplanned caesarean section [n (%)] |  | 80 (24.7) | 14 (28.0) |
| Emergency caesarean section [n (%)] |  | 10 (3.1) | 1 (2.0) |
| PDA [n/N (%)] | 0\|1 | 224/326 (68.7) | 22/50 (44.0) |
| PDA/Vaginal (instrumental) birth mode [n/N (%)] | 0\|1 | 118/208 (56.7) | 14/33 (42.4) |
| Newborn characteristics [n] |  | 333 | 51 |
| Initiate breastfeeding in delivery room* [n/N (%)] | 31\|7 | 286/302 (94.7) | 42/44 (95.5) |
| Postpartum separation* [n/N (%)] | 33\|6 | 52/300 (17.3) | 7/45 (15.6) |
| Sex* [n] | 2\|0 | 331 | 51 |
| Female [n (%)] |  | 157 (47.4) | 27 (52.9) |
| Male [n (%)] |  | 173 (52.3) | 24 (47.1) |
| Divers [n (%)] |  | 1 (0.3) | 0 (0.0) |
| Weight* [n] | 0\|0 | 333 | 51 |
| < 2500 g [n (%)] |  | 6 (1.8) | 1 (2.0) |
| 2500 - 4000 g [n (%)] |  | 293 (88.0) | 46 (90.2) |
| > 4000 g [n (%)] |  | 34 (10.2) | 4 (7.8) |
| Apgar* [n] | 0\|0 | 333 | 51 |
| 5' [median (range)] |  | 10 (5-10) | 10 (5-10) |
| 10' [median (range)] |  | 10 (8-10) | 10 (8-10) |
| * in terms of the number of children (otherwise in terms of the number of mothers)  MD: Missing Data  N: (non)responder in total (without missing data)  PDA: peridural/epidural anaesthesia  BMI: body mass index  Apgar: appearance/ pulse/ grimace/ activity/ respiration | | | |
