# Supplementary material for: How can we support the individual breastfeeding experience? Quantitative results from a mixed-methods study
Source: Int Breastfeed J. 2025 May 17;20:38. doi: 10.1186/s13006-025-00726-4 (PMC12085814; doi:10.1186/s13006-025-00726-4)
Supplement: Supplementary file 3 — Additional file 3: Mother’s breastfeeding attitudes and perceptions by exclusively breastfeeding 4 months and breastfeeding 12 months postpartum. [file 13006_2025_726_MOESM3_ESM.docx]

**Additional file 3** Mother’s breastfeeding attitudes and perceptions by exclusively breastfeeding 4 months and breastfeeding 12 months postpartum

| **Variable** | **Exclusively breastfeeding** | | | | | **Breastfeeding^ep^ 12 months postpartum** | | | | |
| --- | --- | --- | --- | --- | --- | --- | --- | --- | --- | --- |
|  | **< 4 months** | | **≥4 months** | | **p-value** | **No** | | **Yes** | | **p-value** |
|  | MD |  | MD |  |  | MD |  | MD |  |  |
| **Attitude, initiation and sensation regarding breastfeeding [n]** |  | **48** |  | **157** |  |  | **62** |  | **136** |  |
| Motivation to breastfeed | 1 |  | 1 |  | **0.011**^u^ | 1 |  | 1 |  | **0.042**^u^ |
| Strongly agree [n (%)] |  | 41 (87.2) |  | 151 (96.8) |  |  | 55 (90.2) |  | 131 (97.0) |  |
| Agree [n (%)] |  | 5 (10.6) |  | 5 (3.2) |  |  | 5 (8.2) |  | 4 (3.0) |  |
| Neither agree nor disagree [n (%)] |  | 1 (2.1) |  | 0 (0.0) |  |  | 1 (1.6) |  | 0 (0.0) |  |
| Disagree [n (%)] |  | 0 (0.0) |  | 0 (0.0) |  |  | 0 (0.0) |  | 0 (0.0) |  |
| Strongly disagree [n (%)] |  | 0 (0.0) |  | 0 (0.0) |  |  | 0 (0.0) |  | 0 (0.0) |  |
| Planned duration of exclusively breastfeeding | 5 |  | 6 |  | -^cx^ | 4 |  | 4 |  | -^cx^ |
| < 4 months [n (%)] |  | 2 (4.7) |  | 2 (1.3) |  |  | 3 (5.2) |  | 5 (3.8) |  |
| ≥4 until < 7 months [n (%)] |  | 27 (62.8) |  | 105 (69.5) |  |  | 37 (63.8) |  | 87 (65.9) |  |
| ≥ 7 months [n (%)] |  | 14 (32.6) |  | 44 (29.1) |  |  | 18 (31.0) |  | 40 (30.3) |  |
| Breastfeeding counselling in hospital | 4 |  | 2 |  | -^cx^ | 3 |  | 3 |  | -^cx^ |
| Yes, no remaining questions [n (%)] |  | 37 (84.1) |  | 133 (85.8) |  |  | 52 (88.1) |  | 114 (85.7) |  |
| Yes, questions not sufficiently answered [n (%)] |  | 6 (13.6) |  | 18 (11.6) |  |  | 6 (10.2) |  | 17 (12.8) |  |
| No, but I would have liked to talk about it [n (%)] |  | 1 (2.3) |  | 2 (1.3) |  |  | 1 (1.7) |  | 1 (0.8) |  |
| No, no need [n (%)] |  | 0 (0.0) |  | 2 (1.3) |  |  | 0 (0.0) |  | 1 (0.8) |  |
| Actively offered breastfeeding counselling in hospital [n (%)] | 2 | 36 (78.3) | 3 | 133 (86.4) | 0.183^c^ | 1 | 53 (86.9) | 3 | 114 (85.7) | 0.827^u^ |
| Asking questions myself concerning breastfeeding/ feeding in hospital [n (%)] | 2 | 31 (67.4) | 3 | 101 (65.6) | 0.820^c^ | 1 | 36 (59.0) | 3 | 86 (64.7) | 0.450^c^ |
| No opportunity to discuss questions concerning breastfeeding/ feeding in hospital [n (%)] | 2 | 0 (0.0) | 3 | 2 (1.3) | -^cx^ | 1 | 0 (0.0) | 3 | 1 (0.8) | -^cx^ |
| Support of breastfeeding start in hospital | 5 |  | 26 |  | **0.010**^u^ | 11 |  | 16 |  | 0.154^u^ |
| Very good [n (%)] |  | 14 (32.6) |  | 69 (52.7) |  |  | 21 (41.2) |  | 59 (49.2) |  |
| Rather good [n (%)] |  | 11 (25.6) |  | 34 (26.0) |  |  | 8 (15.7) |  | 34 (28.3) |  |
| Neither good nor not good [n (%)] |  | 13 (30.2) |  | 15 (11.5) |  |  | 19 (37.3) |  | 12 (10.0) |  |
| Rather not good [n (%)] |  | 2 (4.7) |  | 12 (9.2) |  |  | 1 (2.0) |  | 11 (9.2) |  |
| Not good at all [n (%)] |  | 3 (7.0) |  | 1 (0.8) |  |  | 2 (3.9) |  | 4 (3.3) |  |
| Comfort concerning breastfeeding/ feeding first days postpartum | 1 |  | 2 |  | **0.008**^u^ | 1 |  | 2 |  | 0.083^u^ |
| Very comfortable [n (%)] |  | 7 (14.9) |  | 42 (27.1) |  |  | 13 (21.3) |  | 37 (27.6) |  |
| Rather comfortable [n (%)] |  | 16 (34.0) |  | 66 (42.6) |  |  | 23 (37.7) |  | 57 (42.5) |  |
| Mediocre [n (%)] |  | 18 (38.3) |  | 36 (23.2) |  |  | 16 (26.2) |  | 33 (24.6) |  |
| Rather not comfortable [n (%)] |  | 4 (8.5) |  | 10 (6.5) |  |  | 7 (11.5) |  | 5 (3.7) |  |
| Not comfortable at all [n (%)] |  | 2 (4.3) |  | 1 (0.6) |  |  | 2 (3.3) |  | 2 (1.5) |  |
| Comfort concerning breastfeeding/ feeding 2 months postpartum | 6 |  | 24 |  | **<0.001**^u^ | 13 |  | 15 |  | 0.105^u^ |
| Very comfortable [n (%)] |  | 8 (19.0) |  | 74 (55.6) |  |  | 18 (36.7) |  | 64 (52.9) |  |
| Rather comfortable [n (%)] |  | 18 (42.9) |  | 45 (33.8) |  |  | 23 (46.9) |  | 38 (31.4) |  |
| Mediocre [n (%)] |  | 14 (33.3) |  | 14 (10.5) |  |  | 6 (12.2) |  | 19 (15.7) |  |
| Rather not comfortable [n (%)] |  | 2 (4.8) |  | 0 (0.0) |  |  | 2 (4.1) |  | 0 (0.0) |  |
| Not comfortable at all [n (%)] |  | 0 (0.0) |  | 0 (0.0) |  |  | 0 (0.0) |  | 0 (0.0) |  |
| **Role of the partner [n]** |  | **48** |  | **157** |  |  | **62** |  | **136** |  |
| Satisfaction with partner involvement in breastfeeding/ feeding | 8 |  | 31 |  | 0.146^u^ | 13 |  | 24 |  | 0.150^u^ |
| Very satisfied [n (%)] |  | 22 (55.0) |  | 46 (36.5) |  |  | 26 (53.1) |  | 41 (36.6) |  |
| Rather satisfied [n (%)] |  | 11 (27.5) |  | 62 (49.2) |  |  | 15 (30.6) |  | 53 (47.3) |  |
| Neither satisfied nor dissatisfied [n (%)] |  | 6 (15.0) |  | 14 (11.1) |  |  | 5 (10.2) |  | 15 (13.4) |  |
| Rather dissatisfied [n (%)] |  | 1 (2.5) |  | 3 (2.4) |  |  | 3 (6.1) |  | 2 (1.8) |  |
| Very dissatisfied [n (%)] |  | 0 (0.0) |  | 1 (0.8) |  |  | 0 (0.0) |  | 1 (0.9) |  |
| Partner's attitude towards breastfeeding | 7 |  | 31 |  | -^cx^ | 13 |  | 23 |  | -^cx^ |
| Good and important [n (%)] |  | 39 (95.1) |  | 123 (97.6) |  |  | 45 (91.8) |  | 110 (97.3) |  |
| No opinion [n (%)] |  | 2 (4.9) |  | 2 (1.6) |  |  | 2 (4.1) |  | 3 (2.7) |  |
| Didn't want me to breastfeed [n (%)] |  | 0 (0.0) |  | 0 (0.0) |  |  | 1 (2.0) |  | 0 (0.0) |  |
| I don't know [n (%)] |  | 0 (0.0) |  | 1 (0.8) |  |  | 1 (2.0) |  | 0 (0.0) |  |
| ^ep^ exclusively or partially breastfeeding  MD: Missing Data (MD concerning exclusively breastfeeding: 121, MD concerning breastfeeding 12 months postpartum: 128)  PDA: peridural/epidural anaesthesia  BMI: body mass index  Apgar: appearance/ pulse/ grimace/ activity/ respiration ^u^ calculated by Mann-Whitney U-test ^c^ calculated by chi-square-test  ^cx^ the chi-square-test cannot be performed due to the sample size with an expected cell frequency < 5 | | | | | | | | | | |
