# Supplementary material for: How can we support the individual breastfeeding experience? Quantitative results from a mixed-methods study
Source: Int Breastfeed J. 2025 May 17;20:38. doi: 10.1186/s13006-025-00726-4 (PMC12085814; doi:10.1186/s13006-025-00726-4)
Supplement: Supplementary file 4 — Additional file 4: Sensation of breastfeeding by nursing mothers over time. Legend: t1 | 2| 3 = 2 | 6| 12 months postpartum asymptotic significance calculated by two-factor variance analysis for ranks according to Friedman: pleasant (t1-t2 0.046, t1-t3 0.802, t2-t3 0.563), enjoyable (t1-t2 1.000, t1-t3 0.802, t2-t3 1.000), practical (t1-t2 0.758, t1-t3 0.529, t2-t3 1.000), time consuming (t1-t2 0.000, t1-t3 0.000, t2-t3 0.847), restricts independence (t1-t2 1.000, t1-t3 1.000, t2-t3 1.000), exhausting (t1-t2 1.000, t1-t3 0.716, t2-t3 0.802), painful (t1-t2 0.002, t1-t3 0.025, t2-t3 1.000), difficult to give up alcohol/smoking (t1-t2 0.381, t1-t3 0.716, t2-t3 1.000). [file 13006_2025_726_MOESM4_ESM.pptx]

## Slide 1
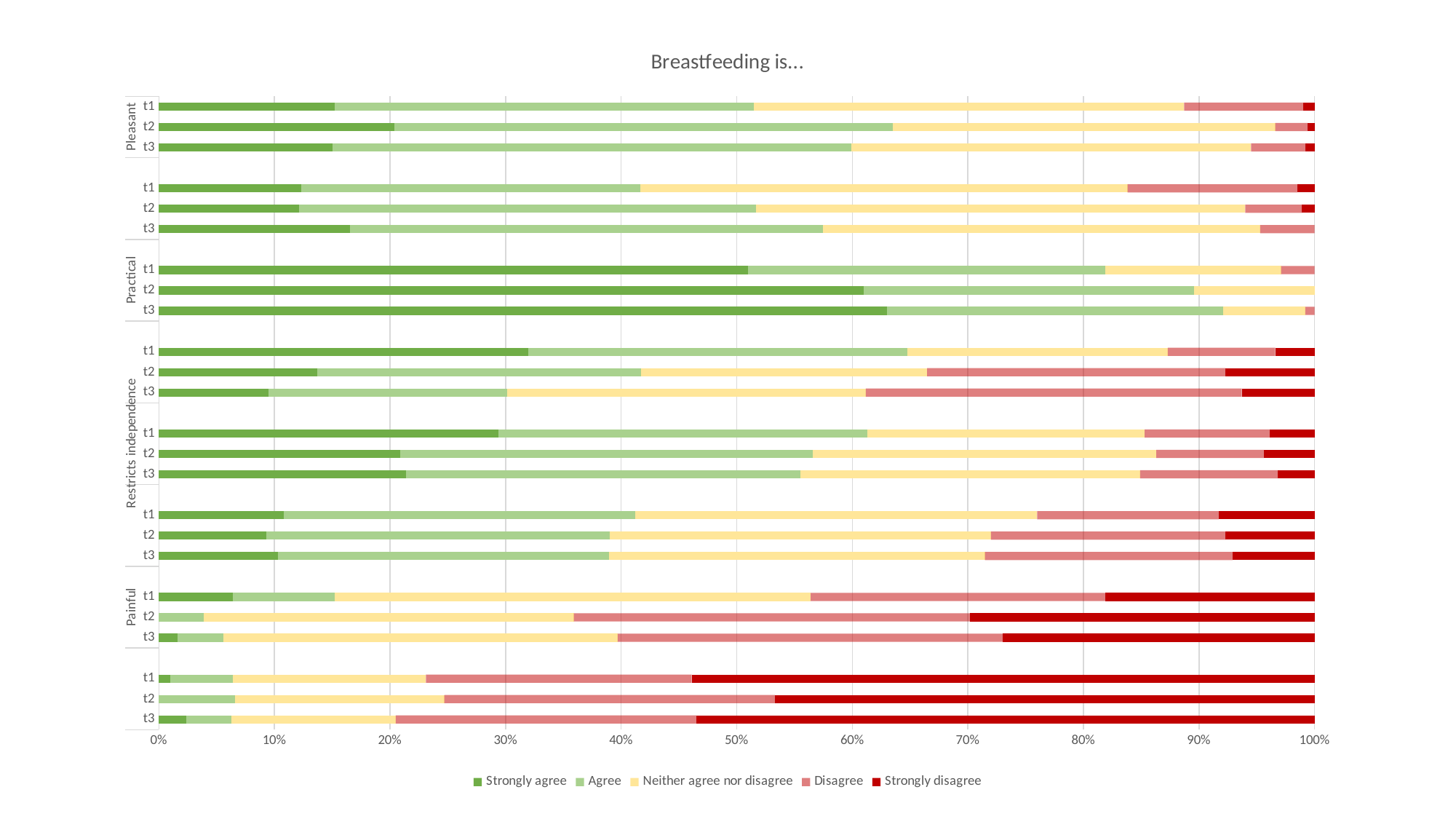

### Chart: Breastfeeding is...
| Category | Strongly agree | Agree | Neither agree nor disagree | Disagree | Strongly disagree |
|---|---|---|---|---|---|
| t3 | 0.024 | 0.039 | 0.142 | 0.26 | 0.535 |
| t2 | 0.0 | 0.066 | 0.181 | 0.286 | 0.467 |
| t1 | 0.01 | 0.054 | 0.167 | 0.23 | 0.539 |
| | None | None | None | None | None |
| t3 | 0.016 | 0.04 | 0.341 | 0.333 | 0.27 |
| t2 | 0.0 | 0.039 | 0.32 | 0.343 | 0.298 |
| t1 | 0.064 | 0.088 | 0.412 | 0.255 | 0.181 |
| | None | None | None | None | None |
| t3 | 0.103 | 0.286 | 0.325 | 0.214 | 0.071 |
| t2 | 0.093 | 0.297 | 0.33 | 0.203 | 0.077 |
| t1 | 0.108 | 0.304 | 0.348 | 0.157 | 0.083 |
| | None | None | None | None | None |
| t3 | 0.214 | 0.341 | 0.294 | 0.119 | 0.032 |
| t2 | 0.209 | 0.357 | 0.297 | 0.093 | 0.044 |
| t1 | 0.294 | 0.319 | 0.24 | 0.108 | 0.039 |
| | None | None | None | None | None |
| t3 | 0.095 | 0.206 | 0.31 | 0.325 | 0.063 |
| t2 | 0.137 | 0.28 | 0.247 | 0.258 | 0.077 |
| t1 | 0.319 | 0.328 | 0.225 | 0.093 | 0.034 |
| | None | None | None | None | None |
| t3 | 0.63 | 0.291 | 0.071 | 0.008 | 0.0 |
| t2 | 0.61 | 0.286 | 0.104 | 0.0 | 0.0 |
| t1 | 0.51 | 0.309 | 0.152 | 0.029 | 0.0 |
| | None | None | None | None | None |
| t3 | 0.165 | 0.409 | 0.378 | 0.047 | 0.0 |
| t2 | 0.121 | 0.396 | 0.423 | 0.049 | 0.011 |
| t1 | 0.123 | 0.294 | 0.422 | 0.147 | 0.015 |
| | None | None | None | None | None |
| t3 | 0.15 | 0.449 | 0.346 | 0.047 | 0.008 |
| t2 | 0.204 | 0.431 | 0.331 | 0.028 | 0.006 |
| t1 | 0.152 | 0.363 | 0.373 | 0.103 | 0.01 |
