# Supplementary material for: How can we support the individual breastfeeding experience? Quantitative results from a mixed-methods study
Source: Int Breastfeed J. 2025 May 17;20:38. doi: 10.1186/s13006-025-00726-4 (PMC12085814; doi:10.1186/s13006-025-00726-4)
Supplement: Supplementary file 5 — Additional file 5: Nursing mothers’ reasons for breastfeeding over time. Legend: t1 | 2| 3 = 2 | 6| 12 months postpartum asymptotic significance calculated by two-factor variance analysis for ranks according to Friedman: natural nutrition (t1-t2 1.000, t1-t3 0.040, t2-t3 0.092), strengthening the bond (t1-t2 1.000, t1-t3 1.000, t2-t3 1.000), supporting child’s health (t1-t2 1.000, t1-t3 0.442, t2-t3 0.765), supporting mother’s health (t1-t2 1.000, t1-t3 0.902, t2-t3 0.442), practical (t1-t2 1.000, t1-t3 1.000, t2-t3 1.000), cheaper (t1-t2 0.951, t1-t3 1.000, t2-t3 0.173), good rhythm between child and mother (t1-t2 0.004, t1-t3 0.043, t2-t3 1.000). [file 13006_2025_726_MOESM5_ESM.pptx]

## Slide 1
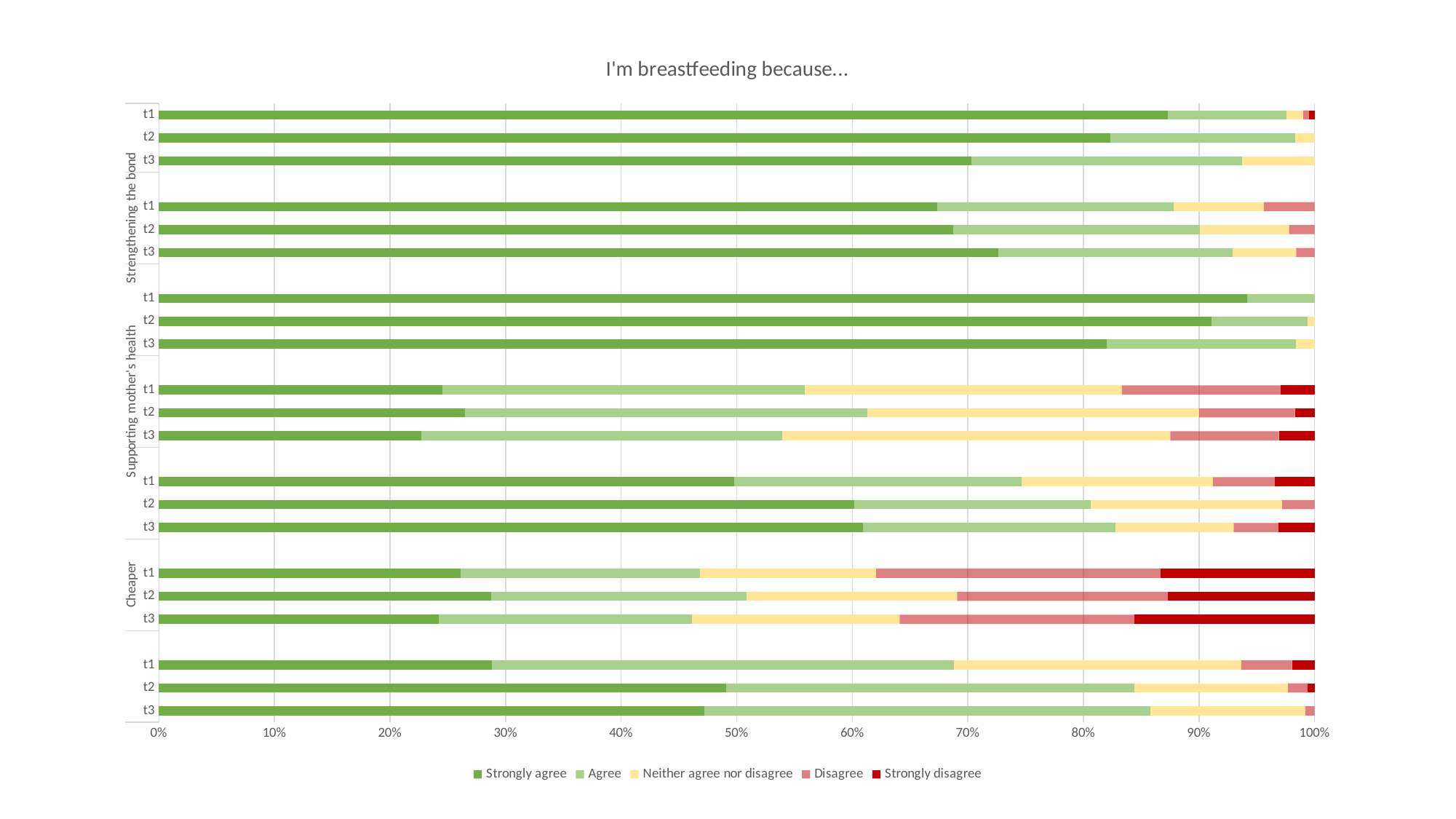

### Chart: I'm breastfeeding because...
| Category | Strongly agree | Agree | Neither agree nor disagree | Disagree | Strongly disagree |
|---|---|---|---|---|---|
| t3 | 0.472 | 0.386 | 0.134 | 0.008 | 0.0 |
| t2 | 0.492 | 0.354 | 0.133 | 0.017 | 0.006 |
| t1 | 0.28780487804878047 | 0.4 | 0.24878048780487805 | 0.04390243902439024 | 0.01951219512195122 |
| | None | None | None | None | None |
| t3 | 0.242 | 0.219 | 0.18 | 0.203 | 0.156 |
| t2 | 0.287 | 0.221 | 0.182 | 0.182 | 0.127 |
| t1 | 0.26108374384236455 | 0.20689655172413793 | 0.15270935960591134 | 0.24630541871921183 | 0.1330049261083744 |
| | None | None | None | None | None |
| t3 | 0.609 | 0.219 | 0.102 | 0.039 | 0.031 |
| t2 | 0.602 | 0.204 | 0.166 | 0.028 | 0.0 |
| t1 | 0.4975609756097561 | 0.24878048780487805 | 0.16585365853658537 | 0.05365853658536585 | 0.03414634146341464 |
| | None | None | None | None | None |
| t3 | 0.227 | 0.313 | 0.336 | 0.094 | 0.031 |
| t2 | 0.265 | 0.348 | 0.287 | 0.083 | 0.017 |
| t1 | 0.24509803921568626 | 0.3137254901960784 | 0.27450980392156865 | 0.13725490196078433 | 0.029411764705882353 |
| | None | None | None | None | None |
| t3 | 0.82 | 0.164 | 0.016 | 0.0 | 0.0 |
| t2 | 0.912 | 0.083 | 0.006 | 0.0 | 0.0 |
| t1 | 0.9414634146341463 | 0.05853658536585366 | 0.0 | 0.0 | 0.0 |
| | None | None | None | None | None |
| t3 | 0.727 | 0.203 | 0.055 | 0.016 | 0.0 |
| t2 | 0.687 | 0.214 | 0.077 | 0.022 | 0.0 |
| t1 | 0.6731707317073171 | 0.2048780487804878 | 0.07804878048780488 | 0.04390243902439024 | 0.0 |
| | None | None | None | None | None |
| t3 | 0.703 | 0.234 | 0.063 | 0.0 | 0.0 |
| t2 | 0.823 | 0.16 | 0.017 | 0.0 | 0.0 |
| t1 | 0.8731707317073171 | 0.1024390243902439 | 0.014634146341463415 | 0.004878048780487805 | 0.004878048780487805 |
