# Supplementary material for: How can we support the individual breastfeeding experience? Quantitative results from a mixed-methods study
Source: Int Breastfeed J. 2025 May 17;20:38. doi: 10.1186/s13006-025-00726-4 (PMC12085814; doi:10.1186/s13006-025-00726-4)
Supplement: Supplementary file 6 — Additional file 6: Mothers'assessments of when breastfeeding counselling would be particularly helpful by exclusively breastfeeding 4 months postpartum. Legend: U2: paediatric examination between 3rd and 10th day of life, U3: in the 4th to 5th week of life, U4: between the 3rd and 4th month of life, U5: between the 6th and 7th month of life. p-value calculated by chi-square-test regarding exclusive breastfeeding ≥ vs. <4 months: registration appointment in clinic 0.067, birth preparation course 0.075, directly after birth 0.510, on the day of birth 0.011, U2 0.425, first days at home 0.001; test cannot be performed due to the sample size with an expected cell frequency < 5 for start of pregnancy|admission to the clinic|U3|in the second month postpartum|U4|U5. [file 13006_2025_726_MOESM6_ESM.pptx]

## Slide 1
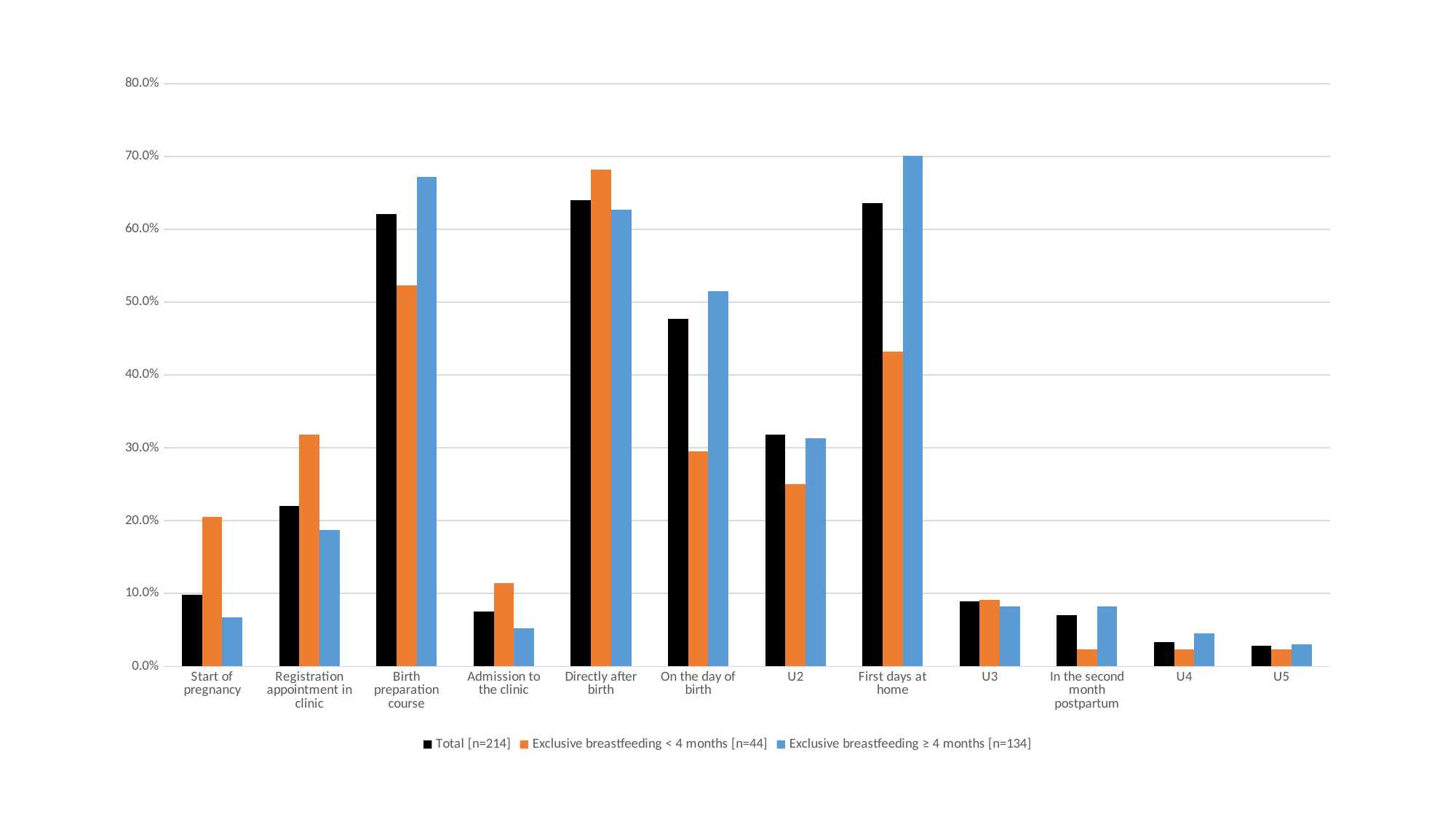

### Chart
| Category | Total [n=214] | Exclusive breastfeeding < 4 months [n=44] | Exclusive breastfeeding ≥ 4 months [n=134] |
|---|---|---|---|
| Start of pregnancy | 0.098 | 0.205 | 0.067 |
| Registration appointment in clinic | 0.22 | 0.318 | 0.187 |
| Birth preparation course | 0.621 | 0.523 | 0.672 |
| Admission to the clinic | 0.075 | 0.114 | 0.052 |
| Directly after birth | 0.64 | 0.682 | 0.627 |
| On the day of birth | 0.477 | 0.295 | 0.515 |
| U2 | 0.318 | 0.25 | 0.313 |
| First days at home | 0.636 | 0.432 | 0.701 |
| U3 | 0.089 | 0.091 | 0.082 |
| In the second month postpartum | 0.07 | 0.023 | 0.082 |
| U4 | 0.033 | 0.023 | 0.045 |
| U5 | 0.028 | 0.023 | 0.03 |
